# Modified “T”-Shaped Umbilicoplasty: A Practical Technique for Creating a Sunken Navel in Abdominoplasty

**DOI:** 10.1093/asjof/ojag002

**Published:** 2026-01-08

**Authors:** Jorge I Borunda

## Abstract

Many umbilicoplasty techniques have been proposed, all with the main goal to creating an aesthetically pleasing navel. In this video article, a “3-flap” umbilicoplasty technique performed by the author is described, in which a T-shaped incision is made in the abdominal wall and limited dissection is used to reposition the navel during abdominoplasty.

**Level of Evidence: 5 (Therapeutic)** 
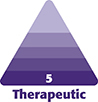

Umbilicoplasty primarily involves the transposition and reinsertion of the navel into the abdominal wall. It is important to ensure that the navel is aesthetically pleasing in terms of size, shape, and position.^1-3^ Craig et al analyzed the characteristics of the female umbilicus and proposed that a small size umbilicus, with a “T”- or vertically shaped and superior hooding was the ideal female umbilicus.^4^ The importance of a deep umbilical depression as a significant factor in the aesthetic appearance of the navel is mentioned in most articles analyzing the navel.^5-7^

To date, many umbilicoplasty techniques have been proposed, all with the main goal of creating a natural-appearing navel and minimizing residual scarring, thereby avoiding any visible signs of a tummy tuck procedure. Although these techniques share certain similarities, they also differ in some key aspects. Notably, the methods vary in how the navel is separated from the umbilical wall, the way the umbilical stalk is attached to the muscle fascia, the types of incisions used for the externalization of the navel, among other aspects, etc.^8-12^

Despite the wide variety of techniques being available for umbilicoplasty, an ideal technique has not yet been established. However, umbilicoplasty techniques that use circular incisions can cause potential complications, including scar contractures and umbilical stenosis.^13,14^ Hence, many surgeons prefer “scarless” methods or approaches involving 2, 3, 4, or multiple flaps.^15-20^

The “3-flap” method described in this study is quick and easy to perform because it requires only a T-shaped incision, a puncture, and a few release cuts to create a tunnel through which the navel will pass. This avoids the need for extensive dissection or fat removal to make the tunnel. In addition, this technique creates a sunken navel (Video).

## SURGICAL TECHNIQUE

This technique involved 2 main steps: (1) detachment of the umbilicus and (2) transposition of the umbilicus in the abdominal wall.

**Figure 1.**
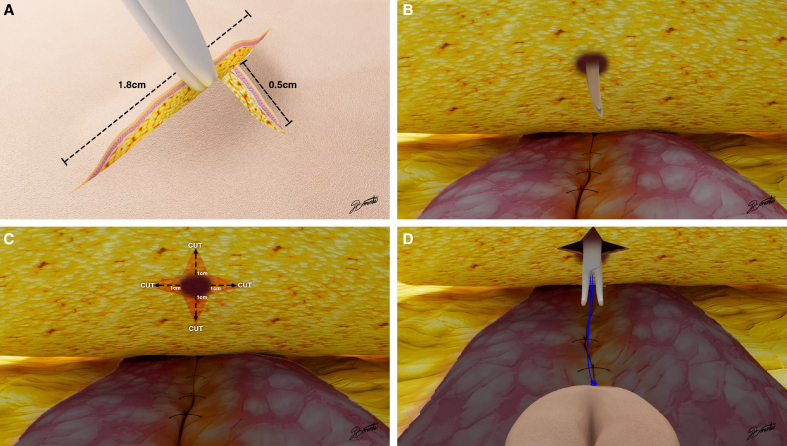
(A) Illustration shows a T-shaped incision, and a straight transfixion puncture performed with scissors through the abdominal wall. (B) Illustration shows the deep area of the abdominal flap and the Metzenbaum scissors pass through the entire flap using a transfixion puncture. (C) Deep flap regions at the level of the T-shaped incision, and cuts of 1 cm made with iris scissors in a cruciform pattern in the fat surrounding the tunnel (dotted lines). (D) The native umbilicus is delivered through the tunnel by gently lifting the suspension sutures with forceps.

**Figure 2.**
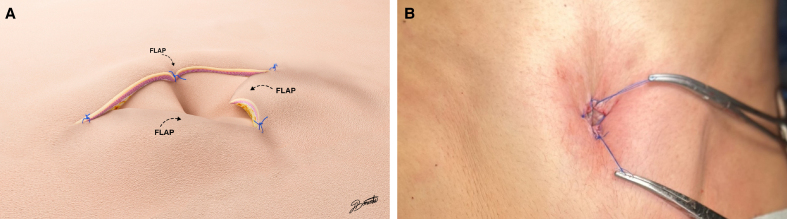
(A) The illustration shows how the edges of the skin flaps surrounding the tunnel turn slightly inward when attached to the navel, giving the navel a sunken appearance and minimizing scar visibility. (B) Intraoperative photograph of a 33-year-old female patient that shows the umbilicus after passing through the tunnel and its insertion into the abdominal wall.

- With the patient under epidural anesthesia, to perform umbilical detachment, 2 suspension sutures were placed on the umbilicus, one at the 12 o’clock position and the other at 6 o’clock. The suture ends were kept long for access.
- Next, we used mosquito forceps to pull the suture ends upward to create adequate tension. A sharp elliptical periumbilical incision was made using a scalpel, leaving a large skin island around the native umbilicus. An electrocautery device was used to carefully dissect the subcutaneous tissue and fascia surrounding the umbilical stalk to ensure complete separation from the abdominal wall.
- Next, navel transposition was performed after completion of abdominal flap dissection, rectus abdominis plicature, and resection of excess abdominal skin.
- Then, to locate the abdominal midline, we drew a line from the xiphoid process to the pubic symphysis. Next, we drew a T-shaped mark on the abdominal flap using a surgical marker, which aligned with or was slightly above the midpoint of the native umbilicus. The length of the horizontal arm of the “T” mark was 1.8 cm and the vertical arm was 0.5 cm.
- A T-shaped incision was made along the premarked lines. Minimal subcutaneous undermining was performed using electrocautery. To ensure maximal preservation, it is important to not remove >1.5 cm^2^ of adipose tissue around the tunnel and using Metzenbaum scissors, we performed a straight transfixion puncture. Next, the abdominal flap was gently everted to visualize the deep flap regions and the fat located around the narrow tunnel created by the puncture (Figure 1A, B). Four 1 cm linear cuts were made in the fat around this tunnel in a cruciform pattern and in the direction of the cardinal axes. This reduced tension and facilitated the navel passage (Figure 1C).
- The native umbilicus was delivered through the tunnel by gently lifting the suspension sutures with forceps (Figure 1D).
- We placed 4 isolated key anchoring sutures to fix the navel to the abdominal skin at the 12, 6, 9, and 3 o’clock positions.
- If required, the umbilicus could be resized or the incision widened to achieve the desired shape.
- Final suturing was then completed to achieve full fixation of the umbilicus to the abdominal wall by performing subcuticular inverted stitches using 4-0 absorbable sutures between the key sutures. The skin flap borders on the abdominal wall surrounding the tunnel rotated slightly inward when fixed to the umbilicus (Figure 2A, B).

After the surgery, a cotton swab soaked in Vaseline was placed inside the navel area and changed at least every 24 h until the stitches were removed. The patient was subsequently instructed to place a small marble wrapped in gauze inside the navel for 3 months to prevent stenosis.

### Patients and Outcomes

From December 2023 to December 2024, 45 tummy tucks with the modified T-shaped umbilicoplasty technique were performed by the author. Forty-five (100%) of the patients were females. The mean age was 38.4 years (range, 18-55 years), and the average follow-up duration was 9.88 ± 2.93 months. The average BMI was 26.74 kg/m^2^.

The overall complication rate was 4.44% (2/45). These complications were minor and not associated with the technique. One of these cases corresponded to a patient who presented a small and partial dehiscence of the umbilicoplasty wound at 5 days postoperatively. This wound dehiscence was ∼5 mm in length and 3 mm in depth and was treated with antibiotic ointment and healed after 2 weeks. The other case involved a patient with hypertrophic scar at the umbilicus who was treated with weekly intralesional triamcinolone acetonide injections. The scar improved after 1 month. There were no cases of umbilical stenosis, navel necrosis, or other complications.

## DISCUSSION

The technique used in this study has several advantages. First, it is a simple and quick technique that requires only 2 small incisions (vertical and horizontal) to form a T-shaped incision creating 3 skin flaps. This avoided the need for skin island resection. Furthermore, the technique involved a limited subcutaneous dissection, a puncture, and 4 release cuts in the cardinal directions, which allowed quick and efficient navel passage through the tunnel to the abdominal wall. Second, the technique is versatile. Once the navel passes through the tunnel, its shape and size can be modified and adjusted according to the surgeon's preference. Excess skin is excised from all 4 quadrants surrounding the navel, permitting precise remodeling and reshaping to achieve a smaller, inverted appearance. On the contrary, if it is necessary to modify the shape of the navel by making it slightly more “elongated,” either vertically or horizontally, this can be achieved by extending the vertical or horizontal branch, respectively, of the T-shaped incision.

**Figure 3.**
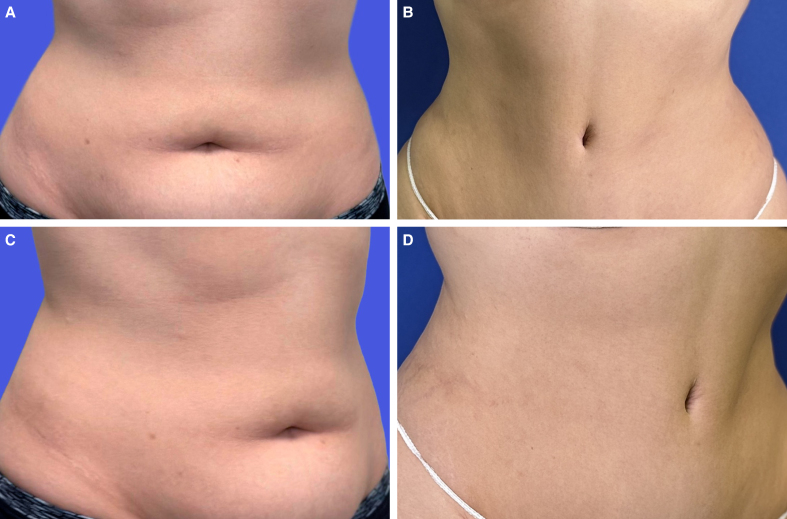
(A) Preoperative photographs of this 33-year-old female patient's belly button before abdominoplasty (frontal view). (B) Postoperative photograph of this 33-year-old female patient's belly button, 18 months after lipoabdominoplasty using the T-shaped umbilicoplasty technique. Here, we can see the sunken navel and how the scar is hidden within the edge of the navel (frontal view). (C) Preoperative photographs of this 33-year-old female patient's belly button before abdominoplasty (lateral view). (D) Postoperative photograph of this 33-year-old female patient's belly button, 18 months after lipoabdominoplasty using the T-shaped umbilicoplasty technique. Here, we can see the sunken navel and how the scar is hidden within the edge of the navel (lateral view).

**Figure 4.**
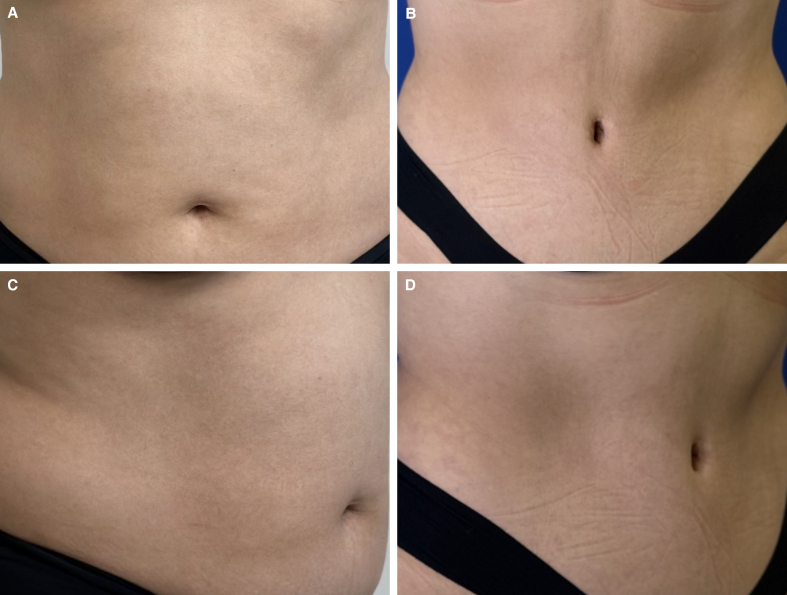
(A) Preoperative photographs of this 39-year-old female patient's belly button before abdominoplasty. The BMI of this patient is 27.5 (frontal view). (B) Postoperative photograph of this 39-year-old female patient's belly button, 28 months after lipoabdominoplasty using the T-shaped umbilicoplasty technique. Here, we can see the sunken navel and how the scar is hidden within the edge of the navel (frontal view). (C) Preoperative photographs of this 54-year-old female patient's belly button before abdominoplasty (lateral view). (D) Postoperative photograph of this 54-year-old female patient's belly button, 8 months after lipoabdominoplasty using the T-shaped umbilicoplasty technique. Here, we can see the sunken navel and how the scar is hidden within the edge of the navel (lateral view).

**Figure 5.**
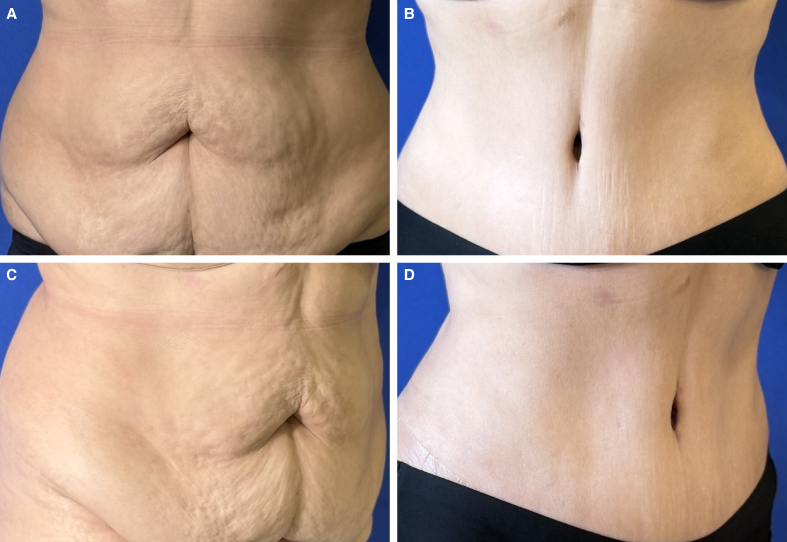
(A) Preoperative photograph of a 54-year-old female patient's belly button before abdominoplasty. The BMI of this patient is 30.2 (Frontal view). (B) Postoperative photograph of the patient's belly button, 8 months after lipoabdominoplasty using the “T”-shaped umbilicoplasty technique. Here we can see the sunken navel and how the scar is hidden within the edge of the navel. (Frontal view). (C) Preoperative photograph of the patient's belly button before abdominoplasty (lateral view). (D) Postoperative photograph of the patient's belly button, 8 months after lipoabdominoplasty using the “T”-shaped umbilicoplasty technique. Here we can see the sunken navel and how the scar is hidden within the edge of the navel. (Lateral view).

Third, like other umbilicoplasty techniques (3- or 4-flap techniques), this technique breaks the scar, avoiding circular scars and reducing umbilical lumen stenosis risk. Furthermore, a key advantage of our technique is the limited dissection and minimal fat removal that occurs during tunnel formation. This allows the fat to be preserved beneath the base of the superior and 2 inferior triangular flaps that surround the tunnel. The fat can then support the base of the flaps, allowing the edges to pivot inward after fixation to the navel. This creates a sunken navel and hides the periumbilical scar within its natural contours. An important detail of our technique is that we can create a sunken navel without having to attach the skin of the native navel to the rectus fascia. We only perform the attachment if the umbilical stalk is very long. Last, our surgical technique gives an aesthetically pleasing navel contour in terms of size and vertical orientation, which makes it attractive for individuals seeking to improve the natural look of their navel (Figures 3-5).

## CONCLUSIONS

In conclusion, this new T-shaped umbilicoplasty technique is safe, reproducible, and does not require a long learning curve. The incision and dissection used to create the tunnel through which the navel is reinserted into the abdominal wall are quick and easy to perform. The incision design and minimal fat removal also influence the creation of an aesthetically sunken navel and offer aesthetic results in terms of the navel's shape and size. This technique is a good alternative for umbilicoplasty and can be confidently performed by all surgeons.
